# Structural finite element model updating considering soil-structure interaction using ls-dyna in loop

**DOI:** 10.1038/s41598-023-31956-3

**Published:** 2023-03-23

**Authors:** Gun Park, Jongwon Jung, Hyungchul Yoon

**Affiliations:** 1 Earthquake Hazards Reduction Center, National Disaster Management Research Institute, Ulsan, 44538 Korea; 2 Department of Civil Engineering, Chungbuk National University, 1 Chungdae-go, Seowon-gu, Cheongju, 28644 Korea

**Keywords:** Civil engineering, Computational science

## Abstract

In this study, a finite element model updating method which can consider soil-structure interaction was developed to analyze the effect of soil properties on the structural response while considering interaction between the soil and the structure. Additionally, LS-DYNA, a commercial finite element program, was included in the loop of the proposed technique using MATLAB to conveniently utilize the complex structures updated by the model. To validate the performance of the proposed method, a large-scale shake table test was conducted. The objective of the validation test was to seek how accurately the proposed model updating method can detect the change in the stiffness. To compare the result of the proposed method with the conventional method, the model updating procedure was conducted with and without considering soil-structure interaction. The proposed finite element model updating method which considers the soil-structure interaction estimated the stiffness of the structure with maximum accuracy of 91%, while the conventional finite element model updating without considering the soil-structure interaction showed maximum accuracy of 88%. By comparing the proposed method with the conventional method without considering the soil-structure interaction, it was confirmed that the proposed method had an 3% higher accuracy on average.

## Introduction

Due to the development of construction techniques and improvements in the quality of construction materials, structures have become higher, larger, and more complex with increased service life. Increasing the service life of structures requires efficient structural maintenance, which has motivated several studies on maintenance techniques. Most maintenance practices are being done manually by inspecting the interior and exterior of a structure, and engineers use inspection results to evaluate the safety. However, these techniques could be subjective and can result differently depending on the level of experience and expertise of the engineer. Therefore, quantitative indicators are required to evaluate the safety of the structure.

Recently, more advanced and automated techniques are being introduced to overcome these limitations. Yoon et al. conducted a study on structural health monitoring using drones and camera equipment. Cha et al. and Narazaki et al. conducted studies to automatically identify cracks in concrete surfaces and automatically recognize structural components using artificial intelligence, respectively. Lee et al. performed a study to automatically extract bridge design parameters using 3D point cloud data^1–4^. However, most of this method was data driven method which do not utilize the finite element (FE) model.

The finite element model can provide an important information related to structurs^5^. However, considering that the FE model is generated using design data, changes due to construction errors or deterioration cannot be considered. Therefore, to generate an accurate FE model that considers the as-is state of the structure, FE model updating methods have been introduced by applying an optimization technique. Jung and Kim proposed a technique for FE model updating using a hybrid genetic algorithm by combining a genetic algorithm and a modified Nelder–Mead algorithm. Cho conducted a study on an automation technique to improve a model that could estimate more realistic and accurate behavior compared to the FE model using the modal coefficients from the natural vibration experiment of high-rise buildings. Gong and Park proposed a technique to construct an inverse eigenvalue problem that could directly obtain the parameters of a FE model from the target mode information and construct a deep neural network to quickly and accurately solve this problem^6–8^.

The current FE model updating methods have two major limitations. First, most of the FE model updating methods have not considered soil-structure interaction effect. In relation to the dynamic behavior of structures, previous studies have revealed that the soil plays an important role^9–12^, and ASCE 4-98 describes the code that must be considered to estimate the dynamic behavior of structures during earthquakes. However, studies on FE model updating that estimate the as-is of a structure using the dynamic behavior of the structure while considering the soil properties are scarce. If the boundary condition of the structure is simplified as a fixed or pin instead of considering soil-properties, the updated FE model will be inaccurate^13–18^. Next, most of the FE model updating methods are not compatible with commercial FE software such as LS-DYNA. LS-DYNA, a commercial FE program, has secured the reliability of the SSI analysis^19–24^; however, it cannot perform efficient FE model updating. On the other hand, it is difficult and time consuming to generate very detailed FE model using programming language such as Java, C, or MATLAB.

Therefore, in this study, we have developed a FE model-updating technique that can consider soil-structure interaction (SSI) effect. The proposed FE model updating system have two major contributions: (1) can consider soil-structure interaction, and (2) can communicate with the commercial software LS-DYNA in the loop. The proposed method considers the SSI effect, so that the updated model is expected to be more accurate and robust to changes in soil properties. Also, the proposed method can communicate with LS-DYNA in loop so that the users can easily use the pre-built FE model together with the developed system.

## System development

Various theories and techniques must be considered when performing the FE model updating of a structure while considering the soil properties. Herein, we describe the FE model updating procedure, theory of the genetic algorithm, and methods of SSI analysis.

### FE model updating procedure

FE model updating is mainly used to obtain information such as the appropriateness of construction for a structure, dynamic properties of the structure, and a FE model considering the as-is state of the structure. FE model updating is mainly performed using dynamic properties obtained from damaged structures^25^. In this study, the FE model was updated using the natural frequency of various structures. Furthermore, to generate a FE model that considers the as-is state of the structure, the element stiffness was set as an unknown variable and the natural frequency estimated by the shaking table test and structural analysis was set as the objective function.

**Figure 1:**
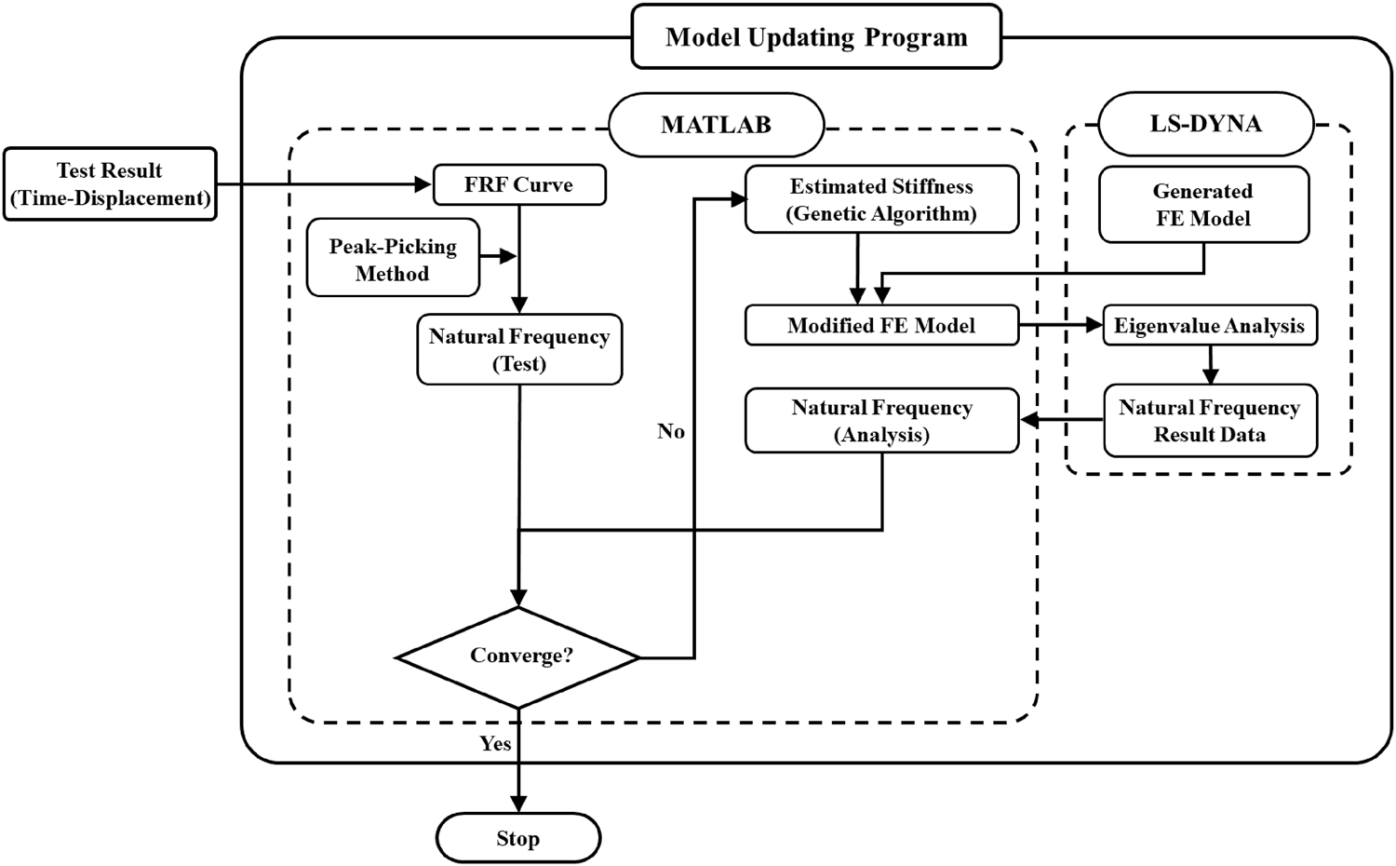
Flowchart for the proposed FE Model updating.

Figure 1 shows the flowchart of the program generated using MATLAB and LS-DYNA for the FE model updating considering the as-is state of the structure. The frequency response function (FRF) curve was generated using the time-displacement data measured by the shaking table test, whereas the natural frequency of the structure was estimated using the peak-picking method. The FE model was generated using LS-DYNA, and a modified FE model was generated by estimating the stiffness with a model-updating program using MATLAB. Furthermore, eigenvalue analysis was performed using LS-DYNA with a modified FE model to estimate the natural frequency. The root mean square (RMS) error was calculated by comparing the natural frequency of the structure estimated using the test and analysis, and the program was terminated if the RMS error was within the error tolerance. Otherwise, the process of estimating the stiffness of the structure using the genetic algorithm and generating the FE model to perform the analysis was repeated.

LS-DYNA generates an input file with file extension of “*.dyn” format, containing nodes, elements and material properties, etc. An eigenvalue analysis was performed with the command prompt through the code generated by MATLAB with this generated input file. LS-DYNA generated an “eigout” file, which contains the eigenvalue of the structure for each mode when the eigenvalue analysis was complied. Only the natural frequency for each mode was scanned among the information in the “eigout” file through MATLAB, and compared with the natural frequency for the test results, and the RMS error was calculated. If the RMS error exceeds the error tolerance, the new wall stiffness was generated using genetic algorithm by MATLAB. The new wall stiffness was used to modify the “*.dyn” format file, which is the input file of LS-DYNA. In order to increase the efficiency when editing the input file, the part related to the wall stiffness was searched, and modified with the new wall stiffness using the genetic algorithm by MATLAB. Eigenvalue analysis was performed again using the command prompt through the modified LS-DYNA input file, and the process of scanning the eigenvalue for each mode was looped in the generated “eigout” file.

### Optmization using genetic algorithm

The genetic algorithm is an optimization technique that uses Charles Darwin's principle of survival of the fittest and Mendel's law of inheritance to find an optimal solution or a similar optimal solution based on biological evolution. Compared to the prior optimization algorithm, the search in the genetic algorithm is performed in parallel with the population formed by the gathering of individuals rather than by the individuals. In addition, global optimization is possible such that the direction or region of search does not excessively depend on the initial value and changes probabilistically depending on the generation^26^. The genetic algorithm expresses the solution to the problem to be solved as a binary vector, and an individual expressed in this method is called a chromosome. Furthermore, the genetic algorithm selects the evaluated population probabilistically or generates a new generation comprising new individuals that are different from the previous generation through genetic manipulation, such as crossbreeding or mutation.

For parents ${p}_{1}$ and ${p}_{2}$ in the crossbreeding of the genetic algorithm, as shown in Eq. (1), ${c}_{1}$ and ${c}_{2}$ can be generated by crossbreeding, as shown in Eq. (2). The variables before and after the crossover variable are considered to be crossed with each other, and the crossbreeding variable can take the properties of both parents at random, as shown in Eq. (3)^27,28^.

1 $$ \begin{aligned} p_{1} & = \left\{ {x_{1}^{{\left( {p_{1} } \right)}} , \cdots ,x_{i - 1}^{{\left( {p_{1} } \right)}} ,x_{i}^{{\left( {p_{1} } \right)}} ,x_{i + 1}^{{\left( {p_{1} } \right)}} , \cdots ,x_{N}^{{\left( {p_{1} } \right)}} } \right\} \\ p_{2} & = \left\{ {x_{1}^{{\left( {p_{2} } \right)}} , \cdots ,x_{i - 1}^{{\left( {p_{2} } \right)}} ,x_{i}^{{\left( {p_{2} } \right)}} ,x_{i + 1}^{{\left( {p_{2} } \right)}} , \cdots ,x_{N}^{{\left( {p_{2} } \right)}} } \right\} \\ \end{aligned} $$

2 $$ \begin{aligned} c_{1} & = \left\{ {x_{1}^{{\left( {p_{1} } \right)}} , \cdots ,x_{i - 1}^{{\left( {p_{1} } \right)}} ,x_{i}^{{\left( {c_{1} } \right)}} ,x_{i + 1}^{{\left( {p_{1} } \right)}} , \cdots ,x_{N}^{{\left( {p_{1} } \right)}} } \right\} \\ c_{2} & = \left\{ {x_{1}^{{\left( {p_{2} } \right)}} , \cdots ,x_{i - 1}^{{\left( {p_{2} } \right)}} ,x_{i}^{{\left( {c_{2} } \right)}} ,x_{i + 1}^{{\left( {p_{2} } \right)}} , \cdots ,x_{N}^{{\left( {p_{2} } \right)}} } \right\} \\ \end{aligned} $$

3 $$ \begin{gathered} x_{i}^{{\left( {c_{1} } \right)}} = x_{i}^{{\left( {p_{1} } \right)}} + \delta \times step_{k} \hfill \\ x_{i}^{{\left( {c_{2} } \right)}} = x_{i}^{{\left( {p_{2} } \right)}} - \delta \times step_{k} \hfill \\ \delta = r_{1} x_{i}^{{\left( {p_{1} } \right)}} - r_{2} x_{i}^{{\left( {p_{2} } \right)}} \hfill \\ r_{1} , r_{2} = R\left( {0,1} \right) \hfill \\ step_{k} = step_{0} \times \left( {\Delta step} \right)^{k} \hfill \\ \end{gathered} $$

Here, $R(\mathrm{0,1})$ is a random number between 0 and 1, and the ∆step can be set after the total generation has passed. In addition, mutations can be considered as follows: If a mutation occurs in the i-th variable after selecting an individual (p), as shown in Eq. (4), the other variables are copied as they are. The corresponding variable generates a random number, as shown in Eq. (6) and considers the number, thereby indicating the mutation in the binary genetic algorithm.

4 $$p=\left\{{x}_{1}^{(p)}, \cdots ,{x}_{i-1}^{\left(p\right)},{x}_{i}^{\left(p\right)},{x}_{i+1}^{\left(p\right)},\cdots ,{x}_{N}^{\left(p\right)}\right\}$$

5 $$c=\left\{{x}_{1}^{(p)}, \cdots ,{x}_{i-1}^{\left(p\right)},{x}_{i}^{\left(c\right)},{x}_{i+1}^{\left(p\right)},\cdots ,{x}_{N}^{\left(p\right)}\right\}$$

6 $${x}_{i}^{\left(c\right)}=\left\{\begin{array}{c}{x}_{i}^{\left(p\right)}+\delta , if \delta \le 0.5 \\ {x}_{i}^{\left(p\right)}-\delta , if \delta >0.5\end{array}\right., \delta =r\times \left(0.5\times {x}_{i}^{\left(p\right)}\right), r=R\left(\mathrm{0,1}\right)$$

In this study, the natural frequency of the structure was used as the objective function that best describes the dynamic properties of the structure. Taking the cross-sectional area and thickness of structure elements as decision variables, the objective function, decision variables and constraint theorems of discrete optimization of the problem are shown in Eq. (7)–9).

7 $$Minimize:W=\sqrt{\sum_{i=1}^{n}\frac{{\left({\overline{\omega }}_{i}-{\omega }_{i}\right)}^{2}}{n}}$$

8 $$Subject to :0<{A}_{obj}\le {A}_{o}$$

9 $$0<{t}_{obj}\le {t}_{o}$$

In Eq. (7–9), ${\overline{\omega }}_{i}$ is the $i$ th natural frequency of the structure of the FE model, and ${\omega }_{i}$ is the $i$ th natural frequency of the structure obtained from the shaking table test. $W$ is the objective function of the genetic algorithm calculated to have the minimum RMS error of the natural frequency of the experiment and analysis. In addition, ${A}_{o}$ is the cross-sectional area of the undamaged wall, ${A}_{obj}$ is the cross-sectional area of the damaged wall, ${t}_{obj}$ is the thickness of the damaged wall, and ${t}_{o}$ is the thickness of the undamaged wall.

### Soil-structure interaction model

**Figure 2:**
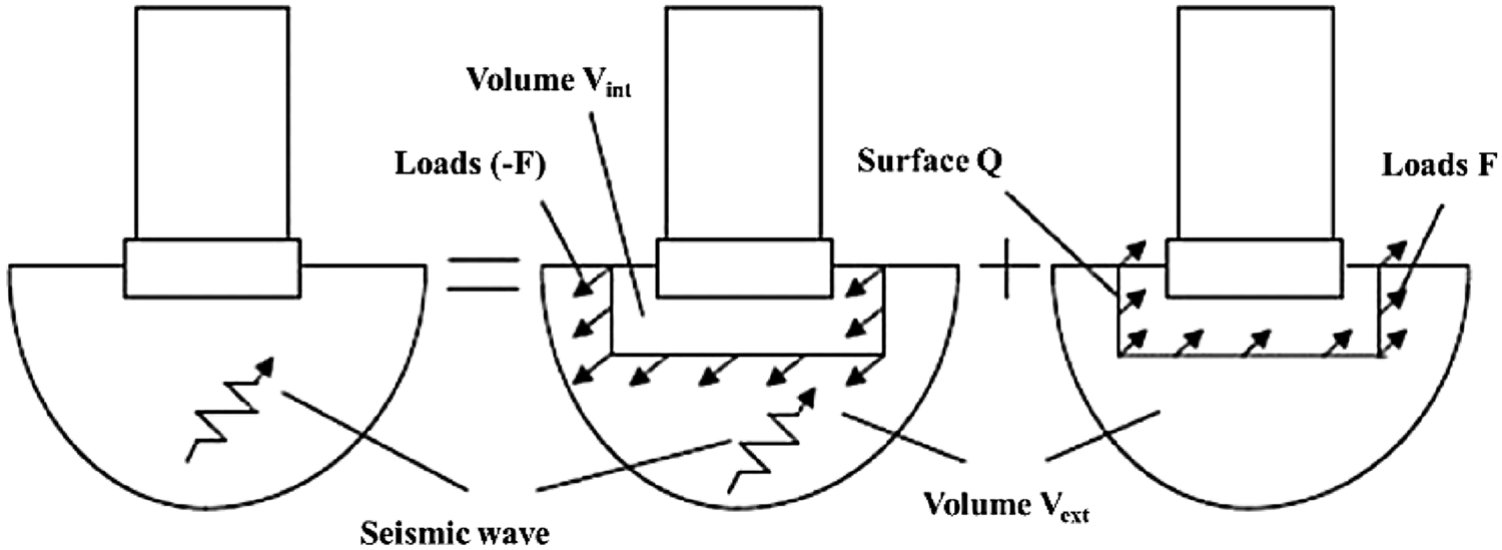
Substructure method for SSI analysis.

**Figure 3:**
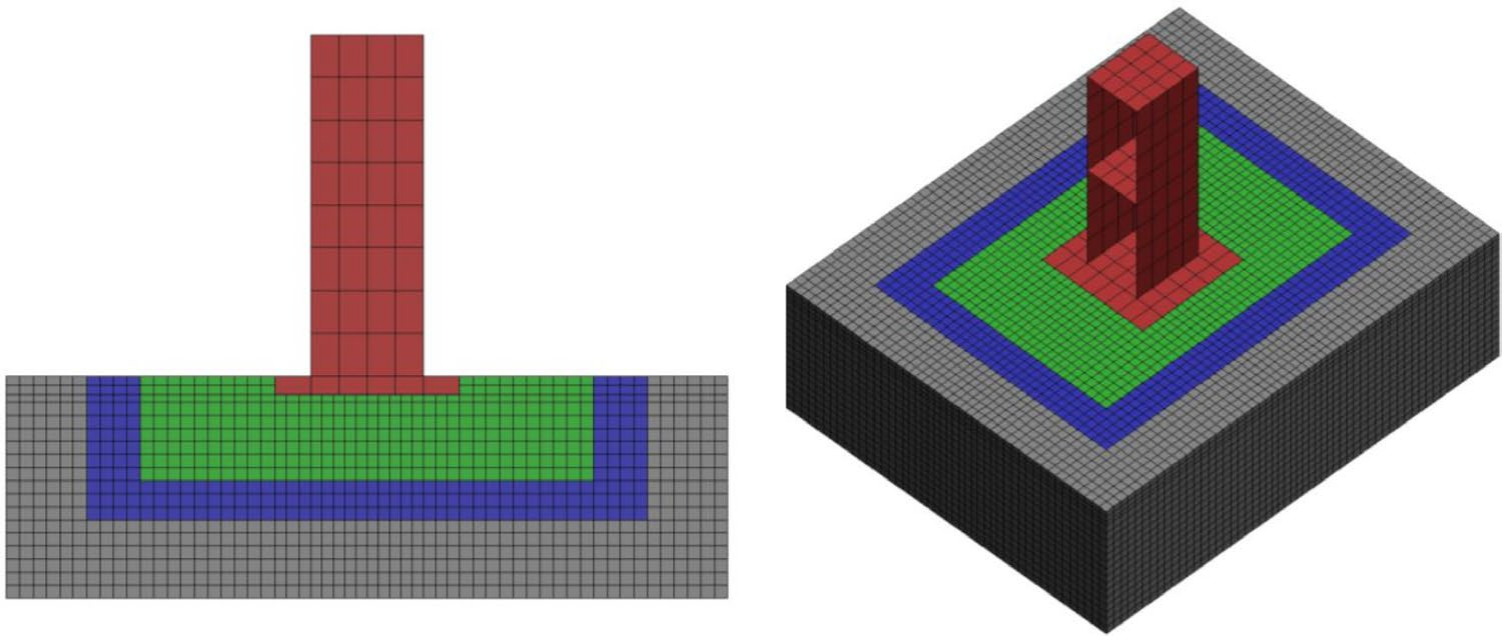
Direct method for SSI analysis^30^.

SSI analysis is a process wherein the structure and soil influence each other's responses under static or dynamic loads. To analyze the SSI problem, the linear/nonlinear behavior of the structure and soil, as well as the nonlinear behavior at the interface between the structure and soil, were analyzed. Various analysis techniques have been proposed to solve the SSI problem, and the most representative methods are the substructure and direct methods, as shown in Figs. 2 and 3, respectively. Although the substructure method can perform a simple analysis compared to the direct method, it is difficult to express the nonlinearity and inhomogeneity of the soil properties. Conversely, the direct method can overcome the nonlinearity between the soil and the structure by directly modeling the nonlinearity and inhomogeneity of the soil properties. However, there are difficulties in modeling the infinite boundary condition and increasing the analysis time. Recently, the direct method tended to be preferred considering that the nonlinear properties of the soil significantly affect the SSI, as revealed by the results of large-scale tests and measurements in many cases^29^. In this study, a FE model of the structure and soil was generated using LS-DYNA, a commercial FE program that uses the direct integration method, and a numerical analysis was performed on the dynamic properties of the structure considering the SSI using this model.

Generating an accurate finite element model using LS-DYNA was important problem. This study modeled the soil using 8-node solid elements based on the study of Bolisetti and Whittaker^30^. The node on each side can be constrained using the *CONSTRAINED_NODE_SET option, which enables the nodes to move together in horizontal and vertical directions. Secondary nonlinearities such as gapping and sliding can be simulated in LS-DYNA using the *CONTACT_AUTOMATIC_SURFACE_TO_SURFACE option^30^.

LS-DYNA has the option of modeling viscous damping (for both soil and structural elements) that is independent of the frequency, unlike Rayleigh damping^30^. This frequency-independent damping can be provided using the *DAMPING_FREQUENCY_RANGE option. A small value of damping (< 2%) should be used as recommended in the LS-DYNA keyword user manual^31^. A damping ratio of 0.02 is specified in the frequency range of 1 to 25 Hz, which is adequate for the analysis presented in this study.

The finite element model used in this study, which does not consider the as-is, was prepared by referring to the prior study by Bolisetti and Whittaker (2015) and a theory on the soil-structure interaction analysis was referring to the prior study by Erfani et al.(2021)^32^ and Forcellini (2021)^33^.

## Validation test

To verify the proposed structural damage estimation method, a shaking table test was performed. By comparing structures with the same damage but located and not located on soil, the change in the dynamic properties of the structure with or without soil, as well as the structure with or without damage was evaluated.

### Test setup

**Figure 4:**
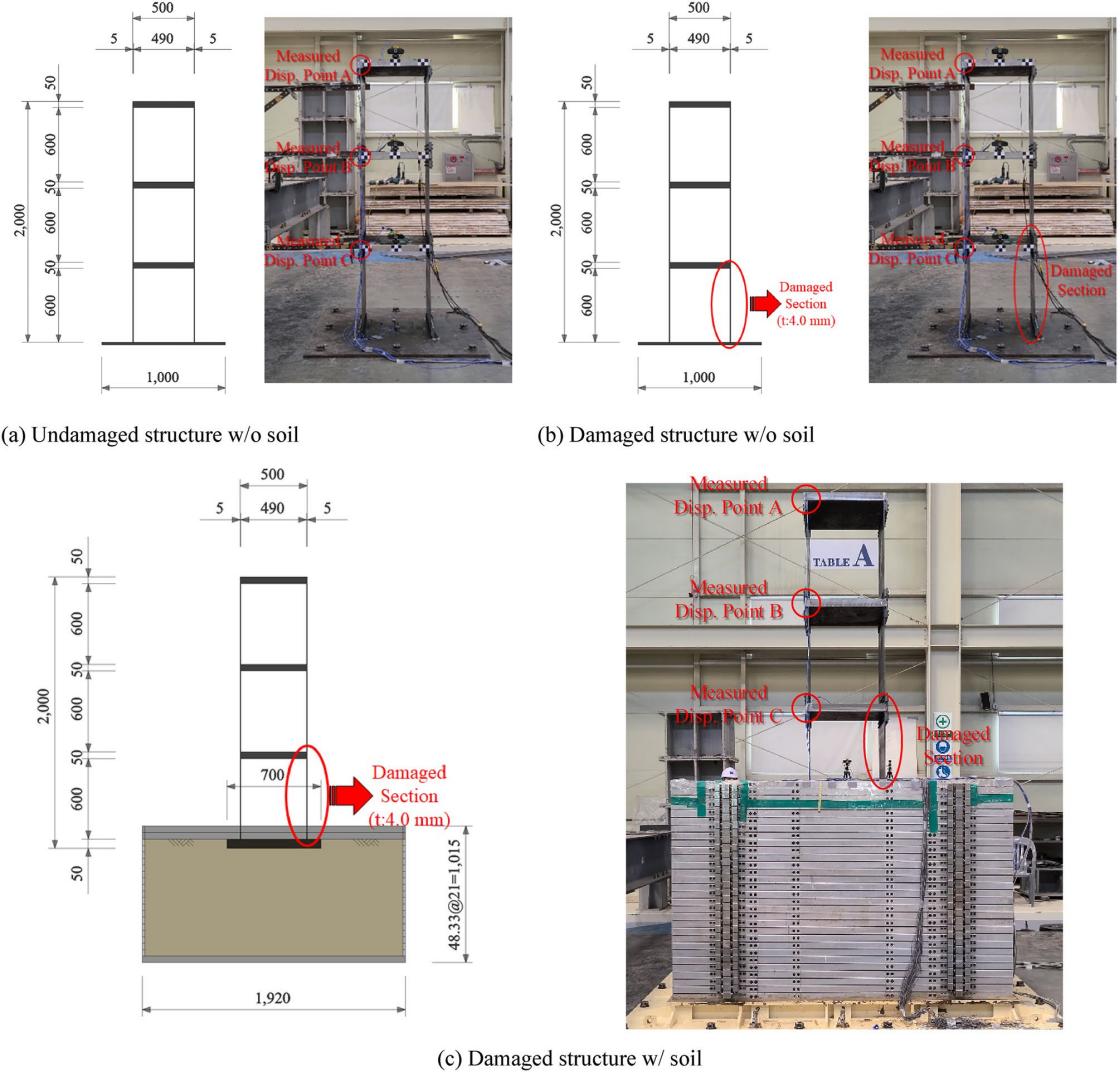
Validation test setup.

A three-story structure with a total height of 2,000 mm and a height by relative story of 600 mm was manufactured using steels. The material properties are listed in Table 1 for the shake table test. As shown in Fig. 4a, the wall thickness of the undamaged structure was 5.0 mm, and a 50.0 mm thick member of the slab was used to prevent deformation during the test. Assuming that the damaged wall of the structure exhibited about 50% decrease in bending stiffness compared to the undamaged wall, a 4.0 mm thick wall was used, as shown in Fig. 4b.

**Table 1:** Properties of material used in the test.

Grade	Elastic modulus (GPa)	Poisson’s ratio	Mass density (kN/m^3^)	Yield strength (MPa)	Tensile strength (MPa)
SS400	200.0	0.3	78.5	215.0	400.0

A soil container with a width of 1,920 mm, length of 1,120 mm, and depth of 1,015 mm was installed to confirm the dynamic properties of the structure when the damaged structure was located on the soil and when it was not. The soil container was manufactured by connecting individual members at a height of approximately 48.33 mm to simulate the behavior of the soil during an actual earthquake, as shown in Fig. 4c; the behavior for each soil height when a seismic load was transmitted to the structure through the soil was independently generated. The process of the shaking table test can be summarized as follows:

· Test Case 1: Undamaged structure without soil;

· Test Case 2: Damaged structure without soil;

· Test Case 3: Damaged structure with soil.

### Test load and soil soil properties

**Figure 5:**
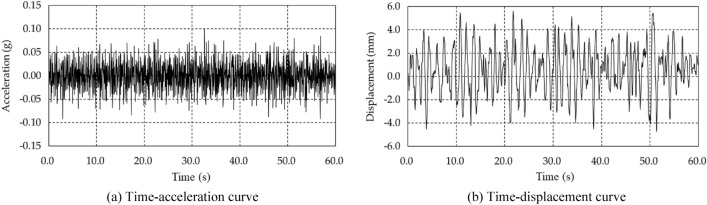
Acceleration acting on the shaking table and measured displacement.

Confirming the dynamic properties of the structure when the shaking table test is performed using seismic loads that have actually occurred is difficult because these loads exhibit unique properties depending on the frequency. Therefore, band limited white noise, which can easily determine the dynamic properties of the structure, was used as the test load, as shown in Fig. 5a,b. The seismic load used in the shaking table test was generated with a total time of 60.0 s and a peaked acceleration of 0.10 g, and the maximum displacement was about 5.0 mm, which was measured from the displacement at the point where the seismic load was applied during the test.

The soil used in the test was sand SP according to the unified soil classification system, and the coefficients of uniformity and curvature were 0.72 and 1.67, respectively. The dry unit weight (γ_d_), maximum dry unit weight (γ_dmax_), and minimum dry unit weight (γ_dmin_) were 16.5, 16.7, and 13.2 kN/m^3^, respectively. Layer compaction was performed every 20 cm by applying a water content of 10.4% in the soil container, and dense sandy soil with a relative density of 95% was generated.

### Test results

**Figure 6:**
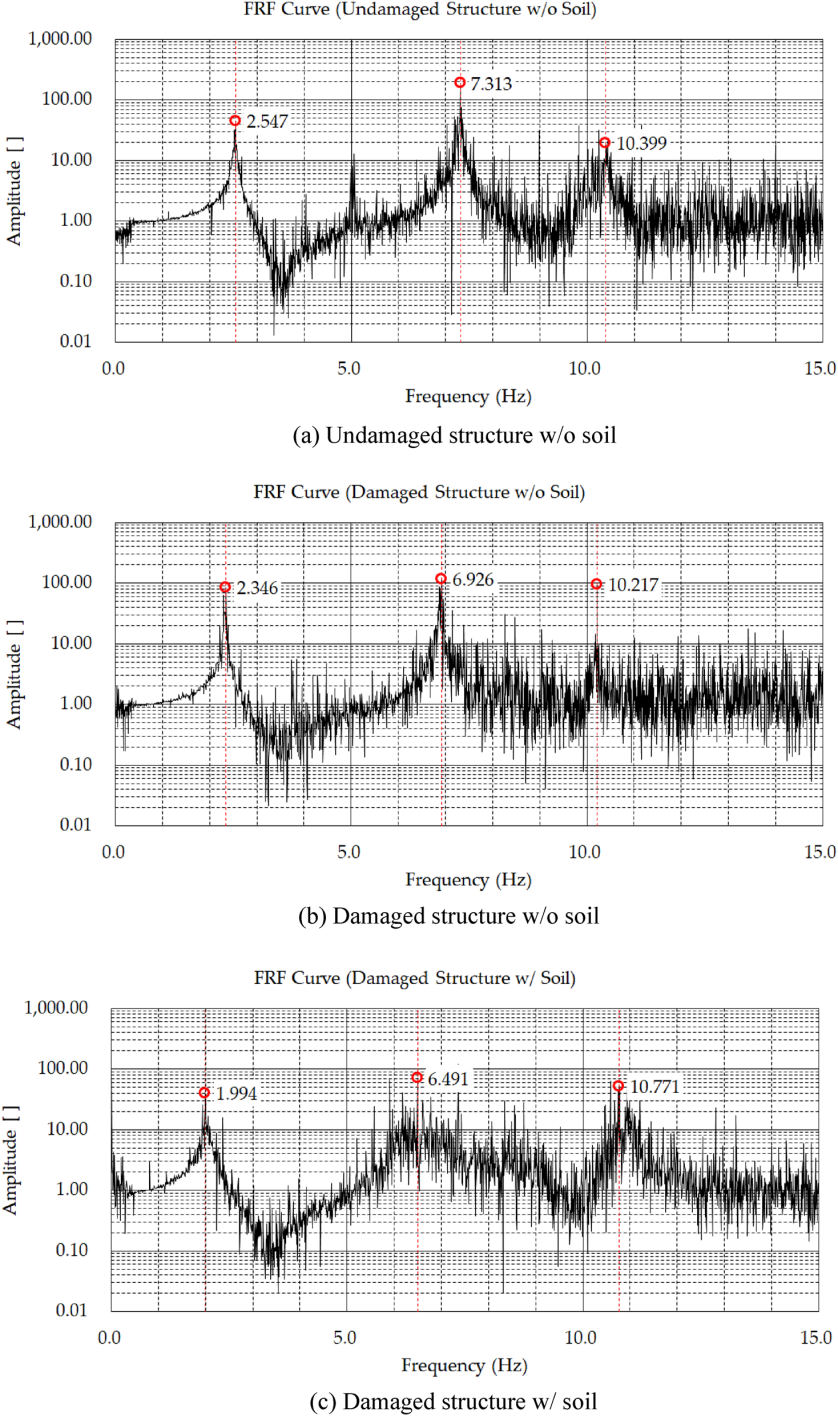
FRF curve.

The displacement data for each floor of the structure was measured using the shaking table test, and a FRF curve was generated based on the results, as shown in Fig. 6. Table 2 presents the estimation of the natural frequency for each test case using the generated FRF curve.

**Table 2:** Natural frequency of structures.

Undamaged structure w/o soil		Damaged structure w/o soil		Damaged structure w/ soil	
Mode	Natural frequency (Hz)	Natural frequency (Hz)	Ratio (%)	Natural frequency (Hz)	Ratio (%)
1st	2.547	2.346	108.58	1.994	117.66
2nd	7.313	6.926	105.59	6.491	106.70
3rd	10.399	10.217	101.78	10.771	94.86

From Fig. 6, it can be observed that the undamaged structure without soil has a larger natural frequency than the damaged structure without soil in the 1st, 2nd, and 3rd modes. In particular, the undamaged structure showed the largest difference of approximately 8% or more compared to the damaged structure in the 1st mode. By comparing the result “w/ soil” with “w/o soil”, the 1st and 2nd natural frequencies increased for 17% and 6% respectively, and the 3rd natural frequency decreased for 6%. The above results confirm that the soil has an impact that cannot be ignored. Therefore, it is necessary to consider the soil when predicting the dynamic properties of the structure.

## FE model updating

To verify the reliability of the FE model updating technique proposed in this study, a FE model was generated and updated using the commercial FE program LS-DYNA. The accuracy of each story and soil stiffness of the structure estimated through model updating were compared with the theoretical stiffness of the material used in the test.

### FE model

**Figure 7:**
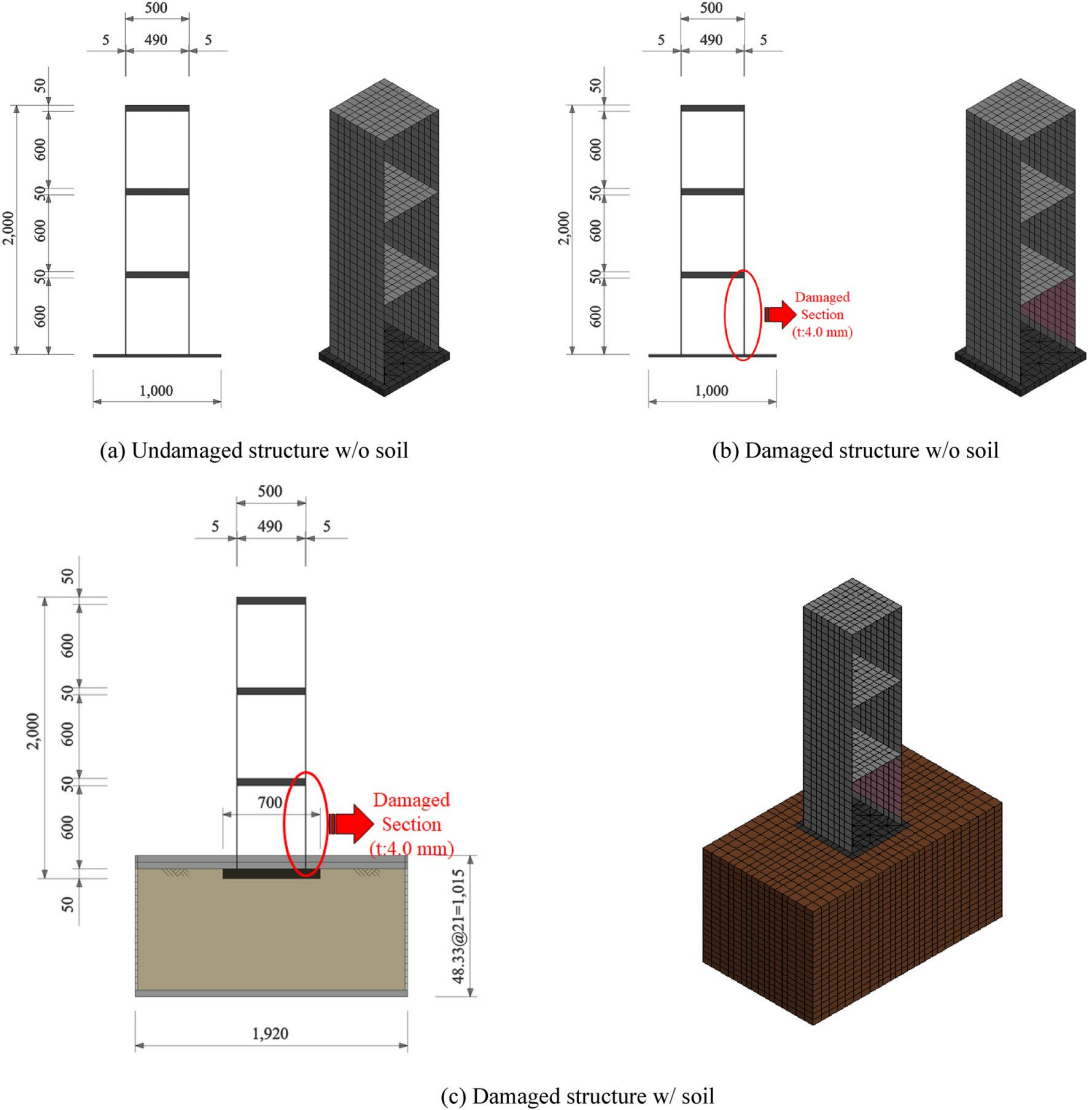
Structure and the FE model used in the validation test.

A FE model with the same specifications as the specimen was generated using a commercial FE program LS-DYNA, as shown in Fig. 7. Four-node shell elements were used for the walls and slab of the structure, and eight-node solid elements were used for the foundation and soil. The slip behavior was simulated at the interface between the structure and the soil by applying a contact element to the interface. In estimating the stiffness of a structure, the material properties, such as the elastic modulus and Poisson's ratio, are assumed to be constant, and only the effective thickness of the wall changes owing to cracks. The elastic modulus of soil is highly likely to change the properties of the physical material as the particles are rearranged by vibration, however, it is assumed that the modulus of elasticity of soil calculated by the initial experiment is maintained in this study. Table 3 shows the properties of the structure and soil FE model generated for the model updating.

**Table 3:** Material properties using FE model.

Type	Elastic modulus	Poisson’s ratio	Unit weight (kN/m^3^)	Material model
Steel	200.00GPa	0.30	78.50	MAT_ELASTIC
Soil	96.0 × 10^3^ kN/m	0.33	16.50	MAT_ELASTIC

### Model updating

We developed a program that could perform model updating in MATLAB using a genetic algorithm. By calculating the natural frequency used as the objective function through LS-DYNA, which is a commercial FE program, we were able to secure the reliability of the results and implement a complex analysis model, as well as consider various material properties. The natural frequency calculated using LS-DYNA was transmitted to the model-updating program generated using MATLAB, and the fitness value was calculated by comparing it with the natural frequency calculated using the test. The fitness value was calculated using the RMS error for each mode of the natural frequencies calculated by the test and the analysis, as in Eq. (7).

7 $$\mathrm{Fitness\  Value}= \sqrt{{({Exp}_{1st}-{Ana}_{1st})}^{2}+{({Exp}_{2nd}-{Ana}_{2nd})}^{2}+{({Exp}_{3rd}-{Ana}_{3rd})}^{2}}$$

**Figure 8:**
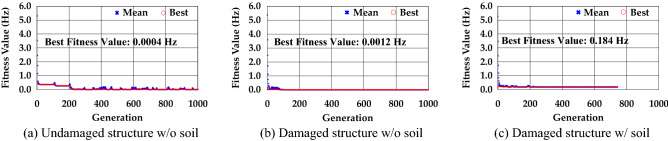
Fitness values at each generation.

Here, ${Exp}_{1st}$, ${Exp}_{2nd}$, and ${Exp}_{3rd}$ are the natural frequencies of the 1st, 2nd, and 3rd modes calculated by the test, respectively. ${Ana}_{1st}$, ${Ana}_{2nd}$, and ${Ana}_{3rd}$ are the natural frequencies of the 1st, 2nd, and 3rd modes calculated using model updating, respectively. The process of finding a solution was performed while narrowing the range of input data applied to variables by increasing or decreasing errors in the model-updating program, as shown in Fig. 8. To prevent the program from calculating indefinitely, the total number of generations was designated as 1,000 and the fitness value was calculated using 100 data points for each generation. The data of the top 40% of the fitness value were transmitted to the next generation, and 40% of the new data were generated within the range of the transmitted data. An additional 20% of the out-of-range mutation data were generated to constitute a new generation. If the fitness value calculated using the newly created generation did not change during the 20th generation, or if the value was 1 × 10^–10^ or less, the program would terminate assuming that it could no longer find the optimal value. The best fitness value was confirmed to be 0.0004 for the undamaged structure without soil, and 0.0012 and 0.184 for the damaged structures without soil and with soil, respectively. Unlike the other two cases, the damaged structure with soil ended after 744 generations considering that there was no change in the fitness value from 724 generations until 20 generations passed.

**Figure 9:**
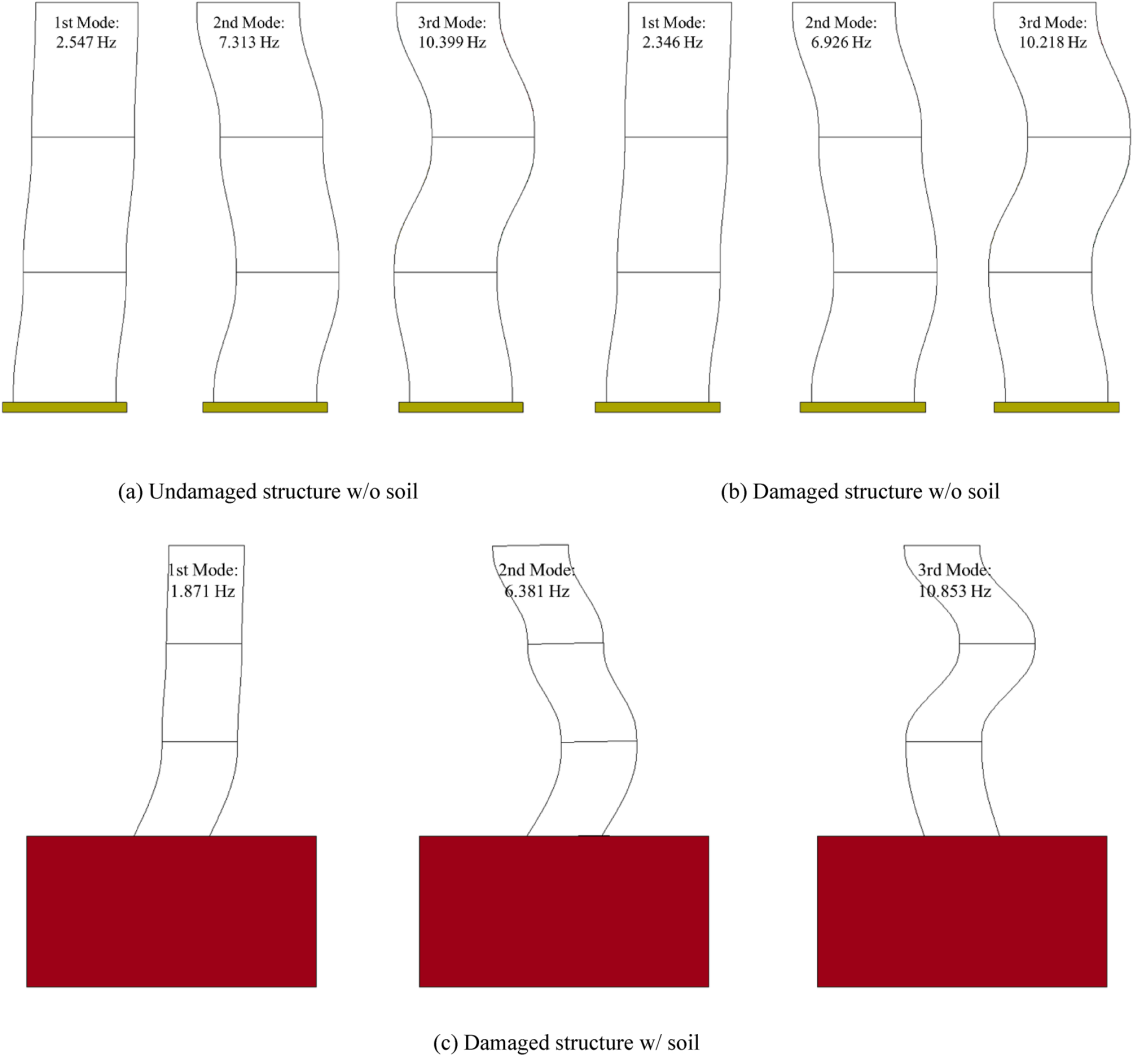
Mode shape estimated by FE model updating.

Table 4 compares the natural frequency of the structure calculated using the test and model updating. The mode shape is shown in Fig. 9. The natural frequencies of the 1st, 2nd, and 3rd modes of the undamaged structure without soil were 2.547, 7.313, 10.399 Hz, and those of the damaged structure without soil were 2.346, 6.926, and 10.218 Hz, respectively. When the estimated natural frequency was compared with the test results, the maximum error in both cases was 0.02%, which confirmed that estimation was highly accurate. The natural frequencies of the 1st, 2nd, 3rd modes of the damaged structure with soil calculated using the model updating were 1.871, 6.3813, and 10.853 Hz, respectively, and a maximum error of 6.2% occurred when compared with the natural frequencies of the test results, which were 1.994, 6.491, and 10.771 Hz, respectively. This is a relatively high error rate compared to the previous cases considering that the material model of the soil with strong nonlinearity was assumed to be elastic.

**Table 4:** Comparison of natural frequencies between model updating and experiment results.

Mode	Undamaged structure w/o soil			Damaged structure w/o soil			Damaged structure w/ soil		
	Analysis (Hz)	Experiment (Hz)	Accuracy (%)	Analysis (Hz)	Experiment (Hz)	Accuracy (%)	Analysis (Hz)	Experiment (Hz)	Accuracy (%)
1st	2.547	2.547	99.98	2.346	2.346	100.02	1.871	1.994	93.83
2nd	7.313	7.313	100.00	6.926	6.926	100.01	6.381	6.491	98.31
3rd	10.399	10.399	100.00	10.218	10.217	100.01	10.853	10.771	100.76

### Estimation of stiffness

Table 5 presents the stiffness of each story estimated using model updating compared to the theoretical stiffness of the structure applied to the test. The accuracy of the estimated stiffness of each story depending on the undamaged or damaged structure without soil ranged within 90% and 97%. Additionally, the damaged structure model with soil showed an accuracy ranging within 83% and 91% in the estimated stiffness for each story. Therefore, the stiffness of the structure can be estimated with an accuracy of over 80%, both with and without damage to the structure as well as with or without soil. Therefore, the proposed model-updating method successfully secured high reliability in estimating the stiffness of the soil and the stiffness of each story of a structure.

**Table 5:** Comparison of floor stiffness between model updating and experiment results.

Type	Undamaged structure w/o soil			Damaged structure w/o soil			Damaged structure w/ soil		
	Analysis (kN/m^3^)	Experiment (kN/m^3^)	Accuracy (%)	Analysis (kN/m^3^)	Experiment (kN/m^3^)	Accuracy (%)	Analysis (kN/m^3^)	Experiment (kN/m^3^)	Accuracy (%)
Floor 1	1.585 × 10^12^	1.667 × 10^12^	95.1	1.351 × 10^12^	1.500 × 10^12^	90.1	1.246 × 10^12^	1.500 × 10^12^	83.1
Floor 2	1.570 × 10^12^	1.667 × 10^12^	94.2	1.520 × 10^12^	1.667 × 10^12^	91.2	1.520 × 10^12^	1.667 × 10^12^	91.2
Floor 3	1.631 × 10^12^	1.667 × 10^12^	97.8	1.581 × 10^12^	1.667 × 10^12^	94.8	1.514 × 10^12^	1.667 × 10^12^	90.8
Average	–	–	95.7	–	–	92.0	–	–	88.4

The general model-updating technique generates a FE model without soil; however, the dynamic properties of the structure used in the objective function were calculated for the real structure while considering the properties of the soil. The objective function was set using the test data of the damaged structure with soil. The FE model applied to the model updating was divided into cases where the soil was considered and where it was not considered, and the stiffness of each story was estimated, as presented in Table 6. In the case of the model with soil, the minimum and maximum accuracies were 83% and 91%, respectively, in estimating the stiffness of each story; however, the model without soil showed a minimum and maximum accuracy of 83% and 88%, respectively. Even for the average estimated structural stiffness, the model that considered the soil showed approximately 3% higher accuracy than the model that did not consider the soil. Therefore, it is confirmed that the soil properties must be considered to improve the reliability of the model updating results, and that the model-updating technique proposed in this study is valid.

**Table 6:** Comparison of floor stiffness according to the FE model applied with model updating.

Type	w/ considering SSI (proposed method) Objective function: Damaged structure w/ soil			w/o considering SSI Objective function: Damaged structure w/ soil		
	Analysis (kN/m^3^)	Experiment (kN/m^3^)	Accuracy (%)	Analysis (kN/m^3^)	Experiment (kN/m^3^)	Accuracy (%)
Floor 1	1.246 × 10^12^	1.500 × 10^12^	83.1	1.251 × 10^12^	1.500 × 10^12^	83.4
Floor 2	1.520 × 10^12^	1.667 × 10^12^	91.2	1.467 × 10^12^	1.667 × 10^12^	87.9
Floor 3	1.514 × 10^12^	1.667 × 10^12^	90.8	1.424 × 10^12^	1.667 × 10^12^	85.4
Average	–	–	88.4	–	–	85.6

## Conclusions

In this study, a new FE model-updating technique that can consider soil-structure interaction was proposed. The proposed method can handle LS-DYNA in the loop which enables the users to easily generate their finite element model. The proposed FE model updating method can optimize not only the stiffness of each member of a structure, but also the stiffness of the soil. To validate the performance of the proposed system, shaking table tests were performed on undamaged structures without soil, damaged structures without soil, and damaged structures with soil. Model updating was performed using the natural frequency of the structure, which was calculated using the shaking table test as the objective function. The stiffness of undamaged and damaged structures without soil were estimated with an accuracy of 95.7% and 92.0%, respectively. The stiffness of the damaged structure with soil was estimated with an accuracy of 88.4%. In addition, by comparing with the model-updating technique without considering the SSI effect, the proposed method showed 3% higher average accuracy.

While the FE model-updating technique proposed in this study was able to estimate the stiffness of a structure with higher accuracy compared to the conventional techniques, there are still some limitations of the proposed method. The proposed model updating could not consider the nonlinearity for both structures and soil, which might be the main source of the error. While the system considers the nonlinearity might need more calculation time, we are planning to consider the non-linearity effect in the future.

In this study, the analysis is performed using the soil stiffness as an initial value, but in future study, we will add a module that estimates the soil stiffness during a seismic load. It is considered that the behavior of the structure can be estimated more accurately by estimating the stiffness of the structure and predicting the change in the properties of the soil when a seismic load occurs.

## Data Availability

The datasets used and/or analysed during the current study available from the corresponding author on reasonable request.
